# Structure of human NTCP reveals the basis of recognition and sodium-driven transport of bile salts into the liver

**DOI:** 10.1038/s41422-022-00680-4

**Published:** 2022-06-20

**Authors:** Hongtao Liu, Rossitza N. Irobalieva, Rose Bang-Sørensen, Kamil Nosol, Somnath Mukherjee, Parth Agrawal, Bruno Stieger, Anthony A. Kossiakoff, Kaspar P. Locher

**Affiliations:** 1 Institute of Molecular Biology and Biophysics, ETH Zürich, Zürich, Switzerland; 2 Department of Biochemistry and Molecular Biology, University of Chicago, Chicago, IL USA; 3 Department of Clinical Pharmacology and Toxicology, University Hospital Zürich, University of Zürich, Zürich, Switzerland

**Keywords:** Cryoelectron microscopy, Mechanisms of disease

Dear Editor,

The sodium taurocholate (TC) co-transporting polypeptide NTCP (SLC10A1) is a secondary active membrane transport protein that mediates the uptake of bile salts from the portal blood plasma into the cytoplasm of liver cells. NTCP was originally cloned from rat and demonstrated to be localized in the basolateral membrane.^1^ Its activity is a key component of bile acid homeostasis and enterohepatic circulation of bile salts (Fig. 1a), a process that includes at least three additional transporter proteins, namely BSEP, ASBT and OSTα/β.^2,3^ In addition to its role in mediating bile salt uptake, NTCP also serves as the receptor of the human hepatitis B and D viruses.^4^ The mechanism by which NTCP recognizes and transports bile salts is not understood, in part because no structural insight into the interaction of NTCP with its substrates is available. While recent studies reported structures of NTCP bound to inhibitory nanobodies^5^ or Fab fragments,^6,7^ no substrate-binding pockets were identified.

**Fig. 1:**
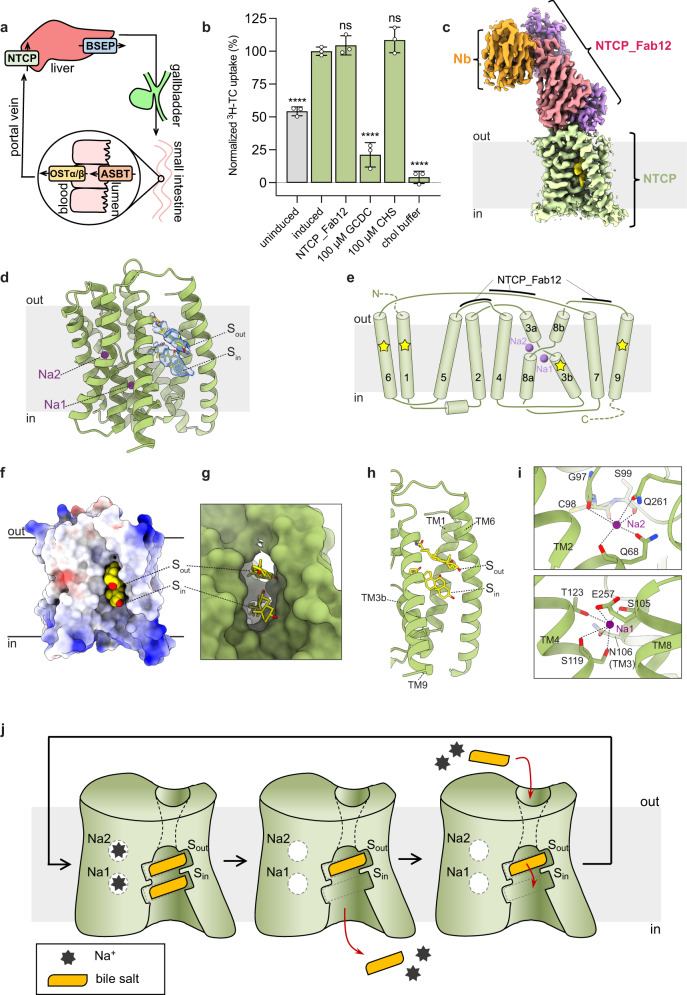
Functional and structural analysis of NTCP. **a** Role of transport proteins in bile acid homeostasis. **b** Transport of radiolabeled TC into HEK293 cells expressing human NTCP fused to a yellow fluorescent protein. Protein expression was induced using doxycycline. Notably, even “uninduced” cells showed a significant level of NTCP expression due to traces of antibiotics in growth media. NTCP_Fab12 was added at a concentration of 750 μM. “Chol buffer” denotes the removal of Na^+^ from the environment and its replacement by choline. Means of three independent replicates are shown; error bars represent the SD. **c** Cryo-EM map of human wild-type NTCP bound to NTCP_Fab12, a Fab-binding nanobody, and substrates. **d** Ribbon diagram of NTCP, with bound Na^+^ ions shown as purple spheres. Bound GCDC molecules are shown as sticks, and the corresponding EM density is shown as a blue surface. **e** Membrane topology of human NTCP. TM helices are numbered. Purple spheres denote the location of bound Na^+^ ions; yellow asterisks denote regions that are in contact with the polycyclic scaffold of bound substrate. **f** Side view of electrostatic surface potential of NTCP, with bound GCDC colored yellow and shown in sphere mode. **g** Close-up view of GCDC-binding pocket, with NTCP shown as a green surface and GCDC as sticks. **h** Close-up view of TM helices clamping the polycyclic scaffold of bound GCDC molecules. **i** Close-up view of sodium binding sites, with contacts shown as dashed lines. **j** Proposed mechanism of NTCP-mediated bile salt transport. See text for explanations.

We expressed wild-type, full-length human NTCP in HEK293 cells and purified the protein in detergent, followed by reconstitution into lipid nanodiscs (Supplementary information, Fig. S1). To facilitate high-resolution structure determination, we isolated Fab fragments from a synthetic library. One such Fab (NTCP_Fab12), bound to the external surface of NTCP with an apparent *K*_d_ value of 60 nM (Supplementary information, Fig. S2). To assess whether NTCP_Fab12 interfered with NTCP function, we measured Na^+^-driven uptake of radiolabeled TC into HEK293 cells. We found that even at a concentration of 0.75 μM (12 times the apparent *K*_d_), NTCP_Fab12 did not interfere with the transport activity of NTCP (Fig. 1b). This suggests that NTCP_Fab12 helped capture human NTCP in a physiologically relevant conformation. In the presence of 100 μM glyco-chenodeoxycholic acid (GCDC), a bona fide substrate of NTCP with a reported *K*_M_ value of ~0.6 μM,^8^ TC uptake was reduced by ~80%.

We determined a 2.9 Å resolution cryo-EM structure of nanodisc-reconstituted NTCP bound to NTCP_Fab12, a Fab-binding nanobody and 100 μM GCDC (Fig. 1c, d; Supplementary information, Figs. S3, S4 and Table S1). The structure revealed a fold that resembled that of the previously reported structures of bacterial homologs of ASBT,^9^ featuring two transmembrane (TM) helices that cross over at the level of the center of the membrane (Fig. 1e). Compared to the bacterial ASBT homolog, NTCP lacks a TM helix (TM1 in bacterial ASBT; Supplementary information, Fig. S5). This has important consequences for the substrate-binding pocket: while bacterial ASBT contains a narrow pocket for a single bile salt molecule that is well-shielded from the lipid bilayer, human NTCP features a cavity that is wide open to the cytoplasm and to the inner leaflet of the bilayer (Fig. 1f).

Our structure revealed a tunnel through the protein that connects the external milieu to the cytoplasm of the hepatocyte and to the inner leaflet of the basolateral membrane (Fig. 1g). The tunnel is a consequence of the separation of TM1 and TM6 from the other TM helices. At the external side, the tunnel is partially open to the outer leaflet of the bilayer and is lined predominantly by hydrophilic residues. In contrast, the cavity exposed to the inner leaflet of the bilayer and the cytoplasm is lined by aliphatic and aromatic side chains, resulting in a hydrophobic surface. As a result, the tunnel has a pronounced polarity with respect to the chemical nature of the protein surface. At its narrowest point, two strong EM density features were visible, revealing two elongated molecules bound to NTCP (Fig. 1f, g). The shapes of these EM densities were consistent with the polycyclic scaffold of bile salts (Supplementary information, Fig. S4). Given that GCDC was present at a concentration that was ~170-fold higher than the reported *K*_M_, we interpreted the observed densities as two bound GCDC molecules. In principle, cholesterol or cholesterylhemisuccinate (CHS), both present during NTCP purification and nanodisc reconstitution, could also be fitted. However, there are no reports of cholesterol being a substrate of NTCP. Furthermore, our functional data demonstrate that CHS did not inhibit NTCP function even at a concentration of 100 μM (Fig. 1b), whereas GCDC showed a pronounced inhibitory effect at this concentration, demonstrating its high affinity for NTCP. The bound substrate molecules are oriented in parallel and clamped between TM1, TM3b, TM6 and TM9 (Fig. 1h; Supplementary information, Fig. S6). Their scaffolds do not appear to be stacked but are rotated by ~90° relative to each other along their long axis. We termed the binding site closer to the external membrane boundary S_out_ and the one closer to the cytoplasm S_in_ (Fig. 1d, f–h; Supplementary information, Fig. S6). The hydrophilic tails of the bound substrate molecules reach inside the aqueous and hydrophilic part of the NTCP tunnel, where they have space to move. Consequently, the glycine moieties of GCDC appear poorly ordered and might adopt multiple conformations, which can rationalize the broad specificity of NTCP for bile salts that contain distinct hydrophilic substituents.

Owing to the high resolution, our EM map revealed density features where sodium ions are expected to bind NTCP based on the structural similarity to the bacterial ASBT homologs.^9^ We identified two sites, designated Na1 and Na2, that are located near the crossing motif of TM3 and TM8 (Fig. 1e). At these sites, bound sodium would be in contact with oxygen atoms from amino acid side chains or main chain carbonyls (Fig. 1i; Supplementary information, Fig. S7). Notably, while most residues interacting with the sodium ions are conserved between the bacterial ASBT homolog and human NTCP,^9^ the site Na2 is shifted by ~4 Å. Our EM map also revealed a density feature approximately halfway between Na1 and Na2 (Supplementary information, Fig. S7). Since no appropriate coordination for bound sodium is present, we assigned this density to a bound water molecule. However, it is conceivable that this site serves as a transient sodium site during the transport cycle.

Our results provide insight into the mechanism of NTCP-mediated bile salt transport. NTCP has been shown to transport two sodium ions with each bile salt molecule.^3^ The coupling of sodium and bile salt transport is consistent with the observation that TM3 and TM8 contribute the binding sites of both sodium ions and bile salts (Supplementary information, Fig. S8). Given the observed transport stoichiometry, only one of the bound bile salt molecules is expected to be transported in a cycle that is powered by the translocation of two sodium ions. The unusual architecture of NTCP featuring an open tunnel is not easily reconciled with a canonical alternating access mechanism invoking occluded conformations, as is observed for many other transport proteins.^10^ Our structural observations suggest an elegant solution to these conceptual challenges and allow us to propose a transport mechanism that can rationalize Na^+^-driven bile salt transport with the experimentally determined stoichiometry of 2 Na^+^:1 bile salt (Fig. 1j). A key feature of the proposed mechanism is that the bile salt molecule at S_in_ is released to the cytoplasm along with the two Na^+^ ions, whereas that at S_out_ remains bound to the transporter, preventing ion leakage. This bile salt is shifted from S_out_ to S_in_ when the transporter is reloading two sodium ions and one bile salt molecule from the external side. Conformational changes are likely required to provide access to the sodium sites Na1 and Na2 from the outside, and for the release of sodium to the inside. These conformational changes, energized by downhill Na^+^ transport along its electrochemical gradient, are thought to be converted into directional displacement of a bile salt from S_out_ to S_in_. The magnitude of the required conformational changes is at present unclear. However, given that NTCP_Fab12 does not inhibit the transport activity of NTCP, it is unlikely that large-scale conformational changes are essential at the external side.

The mechanism that we propose here can also rationalize why NTCP has not been reported to transport cholesterol, which contains a related polycyclic scaffold and is present in significant amounts in the outer leaflet of the basolateral membrane. In the direction of transport (out to in), a cholesterol molecule would have to leave the outer leaflet of the membrane almost completely before entering the aqueous translocation tunnel, which is probably energetically highly unfavorable given the hydrophobicity of cholesterol. In contrast, more hydrophilic bile acids can enter and diffuse through the NTCP tunnel with their polycyclic amphipathic scaffolds first, followed by the hydrophilic head groups, to reach S_out_ in the correct orientation.

In conclusion, our high-resolution structure of human wild-type NTCP reveals the structural basis of bile salt recognition and suggests a transport mechanism. Our results might also provide insight into the human transporters ASBT (SLC10A2, mediating bile salt uptake into intestinal absorptive cells) and SOAT (SLC10A6, facilitating the uptake of sulfated steroids into liver cells). Given the conservation of residues involved in substrate and sodium binding (Supplementary information, Fig. S8), human transporters ASBT and SOAT are likely to share a common transport mechanism with NTCP. Our structure may also serve as a starting point for the development of novel inhibitors that could have therapeutic value to treat cholestasis and related liver diseases or to help prevent attachment of hepatitis B and D viruses to NTCP.^11–13^

## Supplementary information

Supplementary information

## Data Availability

All relevant data are available from the authors and/or included in the manuscript or Supplementary information. Atomic coordinates and EM density maps of human NTCP containing two GCDCs and two Na^+^ ions have been deposited in the Protein Data Bank (7zyi) and the Electron Microscopy Data Bank (EMD-15024).
