# Supplementary material for: Structure of human NTCP reveals the basis of recognition and sodium-driven transport of bile salts into the liver
Source: Cell Res. 2022 Jun 20;32(8):773–6. doi: 10.1038/s41422-022-00680-4 (PMC9343345; doi:10.1038/s41422-022-00680-4)
Supplement: Supplementary file 1 — Supplementary information [file 41422_2022_680_MOESM1_ESM.pdf]

Supplementary Information

Materials and Methods

Protein expression and purification

DNA coding for human wild-type NTCP (Uniprot ID: Q14973) was generated synthetically by GeneArt (Thermo Fisher Scientific) to contain regularly spaced silent restriction sites and codon-optimized for expression in human HEK293 cells. NTCP was expressed as a fusion construct, containing a C-terminal 3C protease cleavage site, preceded by an eYFP-rho-1D4 tag. Stable cell lines for expression were generated using the doxycycline-inducible Flp-In T-Rex system (Thermo Fisher Scientific) according to the manufacturer's guidelines. In addition, a C-terminal Avi-tag was added to the NTCP fusion construct (before the 3C cleavage site) using the PCR method. Similarly, a stable cell line of the Avi-tagged NTCP construct was generated as described above.

Cells were adapted, grown, and maintained in Dulbecco's Modified Eagle Medium (DMEM, Gibco) supplemented with 2% fetal bovine serum (FBS, Thermo Fisher Scientific), 100 units/mL penicillin (Gibco) and 100 µg/mL streptomycin (Gibco) at 37 °C under humidified conditions with 6% CO_2. Gene expression was induced by adding doxycycline (Sigma) at a concentration of 3 µg/mL. Induced cells were maintained for 48 h under the same conditions, followed by harvesting.

All purification steps were performed at 4 °C or on ice, whenever possible. The cell pellet was thawed and resuspended (10:1 vol/wt) in working buffer containing 25 mM Hepes (pH 7.5), 150 mM NaCl, 20% glycerol, supplemented with cOmplete EDTA-free protease inhibitor tablets (Roche) and DNaseI (Roche), and homogenized with a Douncer. n-dodecyl-β-D-maltopyranoside (DDM, Anatrace) and cholesteryl hemisuccinate (CHS, Anatrace) were added to a final concentration of 1% and 0.2% (wt/vol), respectively. Solubilization took place for 2 h with gentle agitation, followed by centrifugation at 100,000×g for 30 min using a Ti-45 rotor (Beckman). Sepharose-coupled Rho-1D4 antibody resin (University of British Columbia) was pre-washed in four rounds, each time with 10 column volumes (CV) of working buffer supplemented with 0.02% DDM and 0.004% CHS (running buffer), prior to batch binding to the supernatant for 2 h at 4 °C. The resin was washed four times with 10 column volumes (CVs) of working buffer. To cleave, the resin was incubated for 2 h with 3 CV of elution buffer (washing buffer supplemented with His-tagged 3C protease, 1:50 wt/wt - 3C protease to the estimated purified protein). The yield of detergent solubilized NTCP is approximately 0.05-0.10 mg per gram of cell pellet. The solution was concentrated using a 50 kDa molecular weight cut-off centrifugal filter (Amicon) before being loaded on a Superdex S200 increase column for size exclusion chromatography. The fractions of the peak containing NTCP were collected and the protein concentration was determined at absorbance of 280 nm using a NanoDrop 2000c spectrophotometer (Thermo Fisher Scientific). Detergent-purified NTCP was further reconstituted into nanodiscs for cryo-EM studies.

Nanodisc reconstitution

A mix of brain polar extract lipids (Avanti Polar Lipids) and cholesterol (Avanti Polar Lipids) (4:1 wt/wt) was solubilized in 1%/0.2% DDM/CHS, followed by sonication. The solubilized lipids were mixed with detergent-purified NTCP and incubated at room temperature for 5 min. Next, membrane scaffold protein (MSP1D1) was added to the mixture and incubated for 20 minutes at room temperature. The molar ratio of the mixture was 1:5:100 (protein:MSP1D1:lipids). Bio-Beads SM-2 (Bio-Rad) were activated with methanol, pre-

equilibrated with HBS buffer (25 mM Hepes (pH 7.5), 150 mM NaCl) and were added to a concentration of 0.8 g/mL to the nanodisc mixture and incubated overnight at 4 °C with gentle stirring. Bio-Beads were removed by passing through a gravity column (Bio-Rad), and the mixture was briefly spun at 4000×g at 4 °C to remove excess lipids. The sample was concentrated using a 50 kDa molecular weight cut-off centrifugal filter (Amicon), followed by size exclusion chromatography ran with HBS buffer. Peak fractions containing nanodisc-reconstituted NTCP were collected and concentrated accordingly.

Cellular uptake assay

Cells stably expressing the NTCP fusion construct with the C-terminal Avi-tag were induced with 3 µg/mL doxycycline and grown for 24 h in supplemented DMEM with 2% fetal bovine serum (FBS, Thermo Fisher Scientific), 100 units/mL penicillin (Gibco) and 100 µg/mL streptomycin (Gibco) under standard conditions. Cells were gently detached and seeded at a density of 150,000 cells per well in a Poly-L-Lysine 24-well plate (Greiner Bio-One). After 2 hours (to aid adherence), the growth media was exchanged with a pre-warmed uptake buffer, consisting of DMEM supplemented with 1 µM sodium taurocholate (TC). The added TC contained a mix of radiolabeled TC (^{3}H-TC) and non-labeled TC in a 1:20 mol/mol ratio. The uptake buffer was further supplemented with 750 nM NTCP_Fab12, 100 µM sodium glyco-chenodeoxycholic (GCDC, Sigma) or 100 µM CHS.

To verify sodium-dependent uptake of TC by NTCP, choline uptake buffer containing 142.9 mM choline chloride, 4.7 mM KCl, 1.2 mM $MgSO_4$, 1.2 mM KH_2PO_4, 1.8 mM $CaCl_2$ and 20 mM HEPES, pH 7.4 was supplemented with 1 µM TC as described above, and used for the uptake assay.

The cells in the uptake buffer were incubated at 37 °C, and the reaction was stopped after 10 minutes by washing the cells twice with ice-cold phosphate buffered saline (PBS, Gibco). Cells were lysed upon addition of 100 µL 1% Triton X-100 in H_2O for 5 minutes. Lysed cells were added to 2 mL scintillation fluid and radioactivity was measured using a scintillation counter (Perkin Elmer 2450 Microbeta2). For assays using choline uptake buffer, cells were washed twice with choline buffer, instead of PBS.

Data were analyzed using GraphPad Prism 8. Background subtracted from all values was calculated by taking the y-intercept of the linear regression through values of TC uptake for induced cells at timepoints between 2 and 20 minutes. Data were normalized to the value of TC uptake under the induced condition. The statistical significance between conditions was determined using Dunnett's test. *p* values are depicted in GraphPad style (GP) as 0.1234 (ns), 0.0332 (*), 0.0021 (**), 0.0002 (***) and <0.0001 (****), and are shown in Fig. 1b.

Enzymatic Biotinylation of NTCP

3C-cleaved NTCP was biotinylated via BirA-mediated biotinylation of the NTCP Avi-tag construct, as described previously[1]. The yield of labeling was verified by a streptavidin pull-down assay.

Phage display

Fab Library E[2], a phage library expressing Fab-fragments on the surface, was used for biopanning (DNA was kindly provided by S. Koide). All five rounds of biopanning were performed in a selection buffer (HBS supplemented with 0.5% bovine serum albumin (BSA) and detergent mixture of 0.02%/0.004% DDM/CHS). In the first round, NTCP was immobilized onto magnetic beads, followed by manual biopanning. Beads were washed three times with

the selection buffer, whereafter only phage expressing Fab-fragments specific to immobilized NTCP remained attached. The beads were resuspended and used to infect log-phase *Escherichia coli* XL-1 Blue cells. Phage were amplified overnight in media containing M13-KO7 helper phage (10^9 pfu/mL) and ampicillin (100 µg/mL) and used as input for subsequent rounds. Four additional rounds of biopanning were performed with decreasing target concentrations. The reduction of nonspecific binders was achieved by preclearing phage pools from each of 2 to 5 rounds with 100 µL streptavidin. The phage pools from the fourth and fifth round were screened for individual clones and validated by a single-point phage ELISA in a 96-well plate (Nunc). Plates were coated with 2 µg/mL neutravidin and blocked with the selection buffer. Individual phage were amplified by inoculating 400 µL media (supplemented with M13-KO7 and 100 µg/mL ampicillin) with *E. coli* XL-1 colonies harboring individual phagemids and incubated overnight in 96-well deep blocks at 37 °C. The supernatant containing the phage particles was collected and diluted tenfold in the selection buffer prior to a phage ELISA assay. Detergent-solubilized NTCP was immobilized on a coated plate at 50 nM concentration for 30 min, followed by 30 min incubation with diluted phage. After 30 min incubation with HRP-conjugated anti-M13 monoclonal antibody (GE Healthcare), TMB substrate (Thermo Fisher Scientific) was added in order to detect signal corresponding to bound phage particles. The selection and ELISA steps were performed at 4 °C.

The clones that bound specifically were sequenced at the University of Chicago Comprehensive Cancer Center DNA Sequencing Facility.

Fab Expression and Purification.

Clones were subcloned into a pRH2.2 plasmid using an In-Fusion Cloning kit (Takara). *E. coli* BL21-Gold cells (Agilent) were transformed using the plasmids expressing the Fab-fragments. Cells were grown in 1 L media supplemented with 100 µg/mL ampicillin to OD_{600} of 0.8, induced with 1 mM IPTG and maintained for a further 4 h at 37 °C. The cells were harvested and stored at -80 °C. The antigen-binding fragments were purified by Protein A chromatography, followed by ion-exchange chromatography as described previously[3].

To estimate the NTCP:Fab binding affinity, a protein ELISA was conducted. Briefly, the same neutravidin-coated 96-well plates were used to immobilize 50 nM detergent-solubilized NTCP. Fab-fragments were subsequently diluted and assayed against the immobilized protein. Binder detection was done using HRP-conjugated mouse anti-human IgG F(ab′)2 (Jackson) and TMB substrate (Thermo Fisher Scientific). The results were plotted and, for the NTCP_Fab12, which was used for structure determination, presented in Fig. S2.

Fab-binding Nanobody Expression and Purification

Fab-binding nanobody[4], fusing one N-terminal His-tag and TEV protease cleavage site in pET26b (+) vector, was transformed into *E. coli* BL21 (DE3) cells for expression with Lysogeny broth medium. The cells were grown to OD_{600} of 0.8 at 37 °C, and induced by addition of 1 mM IPTG and grown for 20 hrs at 20 °C. The cells were harvested and periplasmic protein was obtained via osmotic shock by sucrose gradient. The lysate was purified by Ni-NTA chromatography, and the His-tag was removed with TEV protease followed by a Ni-NTA chromatography.

EM Sample Preparation

Nanodisc-reconstituted NTCP was prepared as described above, and Fab fragments (NTCP_Fab12) and Fab-binding nanobody[4] were added to the sample with 1:1:1.2 molar ratio, before the final SEC. SEC-purified human NTCP in a complex with NTCP_Fab12 and a Fab-

binding nanobody, at a concentration of ~0.7 mg/mL, was equilibrated with 100 μM sodium glyco-chenodeoxycholic (GCDC, Sigma) for 5 min. Samples were applied onto glow discharged Quantifoil R1.2/1.3 carbon/copper 300 mesh grids, followed by plunge freezing in liquid ethane/propane mixture using Vitrobot Mark IV (FEI) at 4 °C and 100% humidity.

EM Data Acquisition and Processing

Data were collected semi-automatically with EPU2 (Thermo Fisher Scientific) on a Titan Krios 300 kV microscope (Thermo Fisher Scientific) equipped with a Gatan K3 camera and a Gatan Biocontinuum energy filter. Image stacks (40 frames) were collected at nominal magnification of 130,000k and corresponding pixel size of 0.33 Å/pix (super-resolution mode). The defocus was in the range of -0.6 to -2 μm, flux was set to 15 e^-/pix/s and total dose was 64 e^-/Å^2 We collected a total of 13,208 multiframe micrographs.

Data processing details are presented in Fig. S3. In short, the multiframe micrographs were imported into RELION 4.0[5], motion corrected with MotionCor 2[6] and binned by a factor of 2 to a pixel size of 0.66 Å/pix. Contrast Transfer Function (CTF) parameters were estimated with Gctf[7]. Particles were picked using the reference-free Laplacian-of-Gaussian routine. Binned particles (factor of 4) were extracted and subjected to several rounds of 2D classification. The best classes were then used to generate an initial model *ab initio* and were further subjected to several rounds of 3D classification. The best particles were used for an initial 3D refinement. The particles were then unbinned (to 0.66 Å/pix) and subjected to several additional rounds of 3D refinement and classification. The final set of particles (161,093) were CTF refined and polished prior to a last round of 3D refinement (with a mask that excluded the density for the nanodisc and the constant domain of NTCP_Fab12) and post processed (with B-factor set to -52 Å^2 and *ad-hoc* low-pass filter to 2.7 Å). The final EM density map had an overall resolution of 2.88 Å, as estimated by the 0.143 Fourier Shell Correlation criterion. Local resolution estimation was performed in RELION and the results are shown in Fig. S3.

Model Building and Refinement

Model building was performed in Coot[8]. The final density map featured well-resolved TM helices (Fig. S4) that allowed for *de novo* model building based on the protein sequence. The N-terminal (residues 1-18) and C-terminal (312-349) are highly flexible and were not resolved. Refinement of the models was performed in Phenix[9] and validation in MolProbity[10] and presented in Table S1. The atomic coordinates and geometrical restraint of the ligands (cholesterol and GCDC) were used from the ligand library (ligand codes: CLR, CHO).

Figure Preparation

Graph preparation and data analysis were performed in GraphPad Prism version 8.0.0 for Windows, GraphPad Software, La Jolla California USA, www.graphpad.com. The images of models and the EM density map were prepared in UCSF Chimera[11] and UCSF ChimeraX[12].

Supplementary References

1. Fairhead, M. & Howarth, M. *Methods Mol Biol* **1266**: 171-184 (2015)
2. Fellouse, F., Wiesmann, C. & Sidhu, S. *Proc Natl Acad of Sci* **101**: 12467-12472 (2004)
3. Kim, J. et al. *Nature* **576**: 315-320 (2019)

4. Ereño-Orbea, J. et al. *J. Mol. Biol.* **430:** 322-336 (2018)
5. Kimanius, D., Dong, L., Sharov, G., Nakane, T. & Scheres, S.H.W. *Biochem J* **478**: 4169-4185 (2021)
6. Zheng, S. et al. *Nature Methods* **14**: 331-332 (2017)
7. Zhang, K. *Journal of Structural Biology* **193**: 1-12 (2016)
8. Emsley, P., Lohkamp, B., Scott, W.G. & Cowtan, K. *Acta Crystallogr D Biol Crystallogr.* **66**: 486-501 (2010)
9. Liebschner, D. et al. *Acta Crystallogr D Struct Biol.* **75**: 861-877 (2019)
10. Williams, C.J. et al. *Protein Sci.* **27**: 293-315 (2018)
11. Pettersen, E.F. et al. *J. Comput. Chem.* **25**: 1605-1612 (2004)
12. Pettersen, E.F. et al. *Protein Sci.* **30:** 70-82 (2021)

Supplementary Information, Figure S1

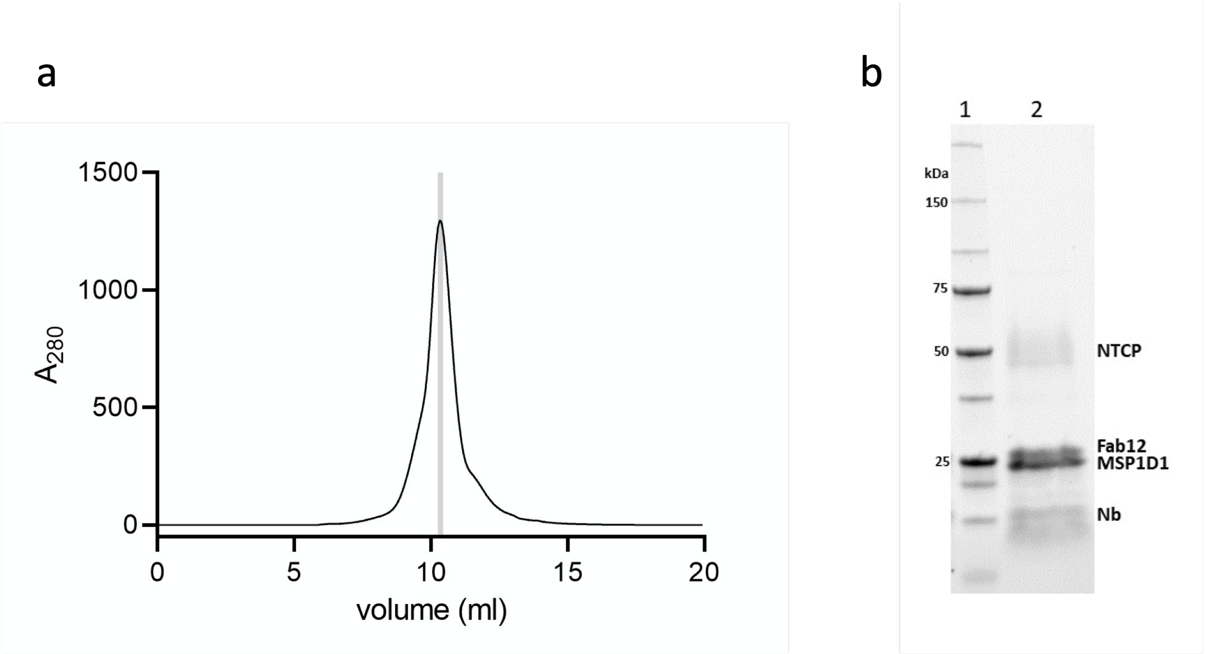

Fig. S1. NTCP purification and nanodisc reconstitution. Size exclusion chromatography and SDS-PAGE analysis of purified nanodisc-reconstituted NTCP in a complex with NTCP_Fab12 and a nanobody. **a,** Size exclusion profile. **b,** SDS-PAGE gel. Lane 1: marker, lane 2: purified complex in nanodiscs.

Supplementary Information, Figure S2

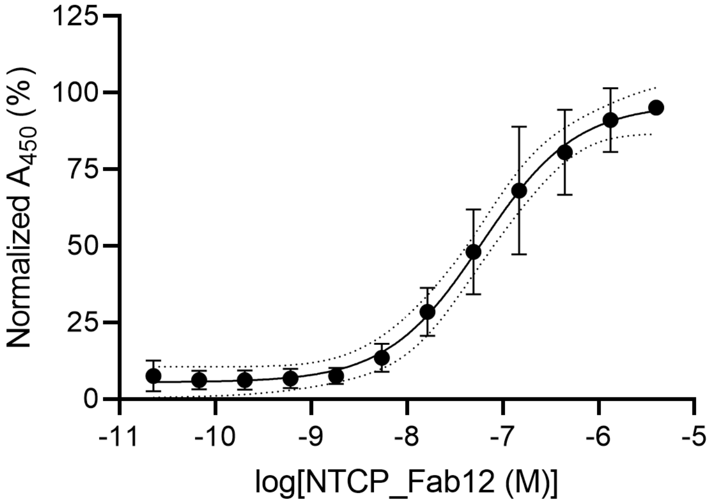

Fig. S2. **Normalized multi-point ELISA for purified NTCP_Fab12.** Calculation of EC_{50} for NTCP_Fab12 binding to nanodisc-reconstituted NTCP. The estimated binding affinity is 60 nM. Data points represent the mean, error bars indicate the standard deviation of three independent measurements. Dotted lines indicate the 95% confidence intervals.

Supplementary Information, Figure S3

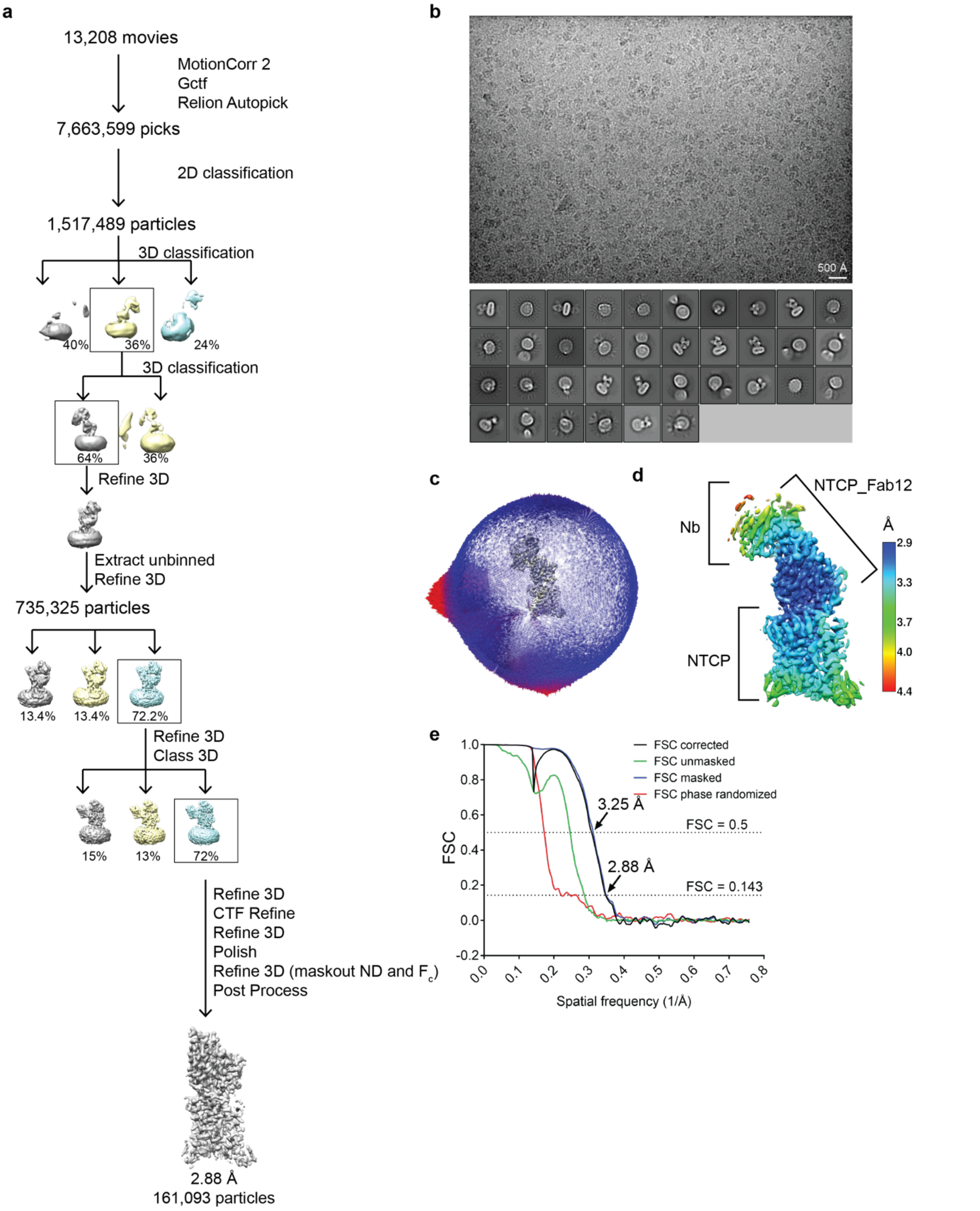

Fig. S3. Cryo-EM data processing of the NTCP:NTCP_Fab12:Nb complex. a, Flowchart of data processing using RELION 4. **b,** Representative micrograph and 2D class averages. **c,** Angular distribution of the particles from the final 3D refinement. **d,** Local resolution estimates as calculated in RELION. **e,** Fourier Shell Correlation as reported in RELION 4.

Supplementary Information, Figure S4

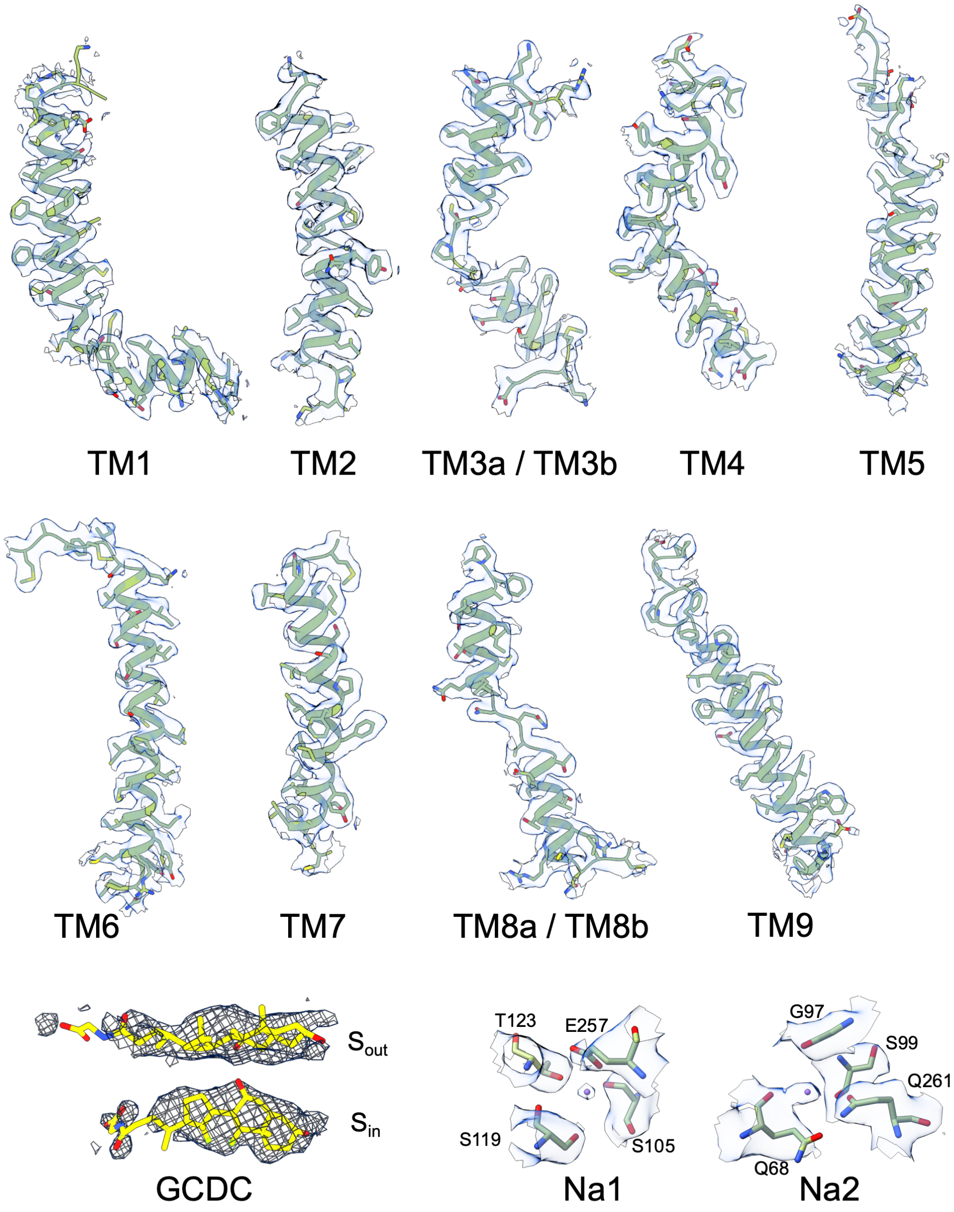

Fig. S4. Detailed EM density maps. EM densities of transmembrane (TM) segments and substrate binding sites of human NTCP. The contour level is set to the same threshold throughout the figure. The protein is shown in mixed stick and ribbon representation. The EM density is shown as a surface except for bound substrate (GCDC molecules), where the EM density is shown as a dark mesh. Presumed sodium ions are shown as purple spheres.

Supplementary Information, Figure S5

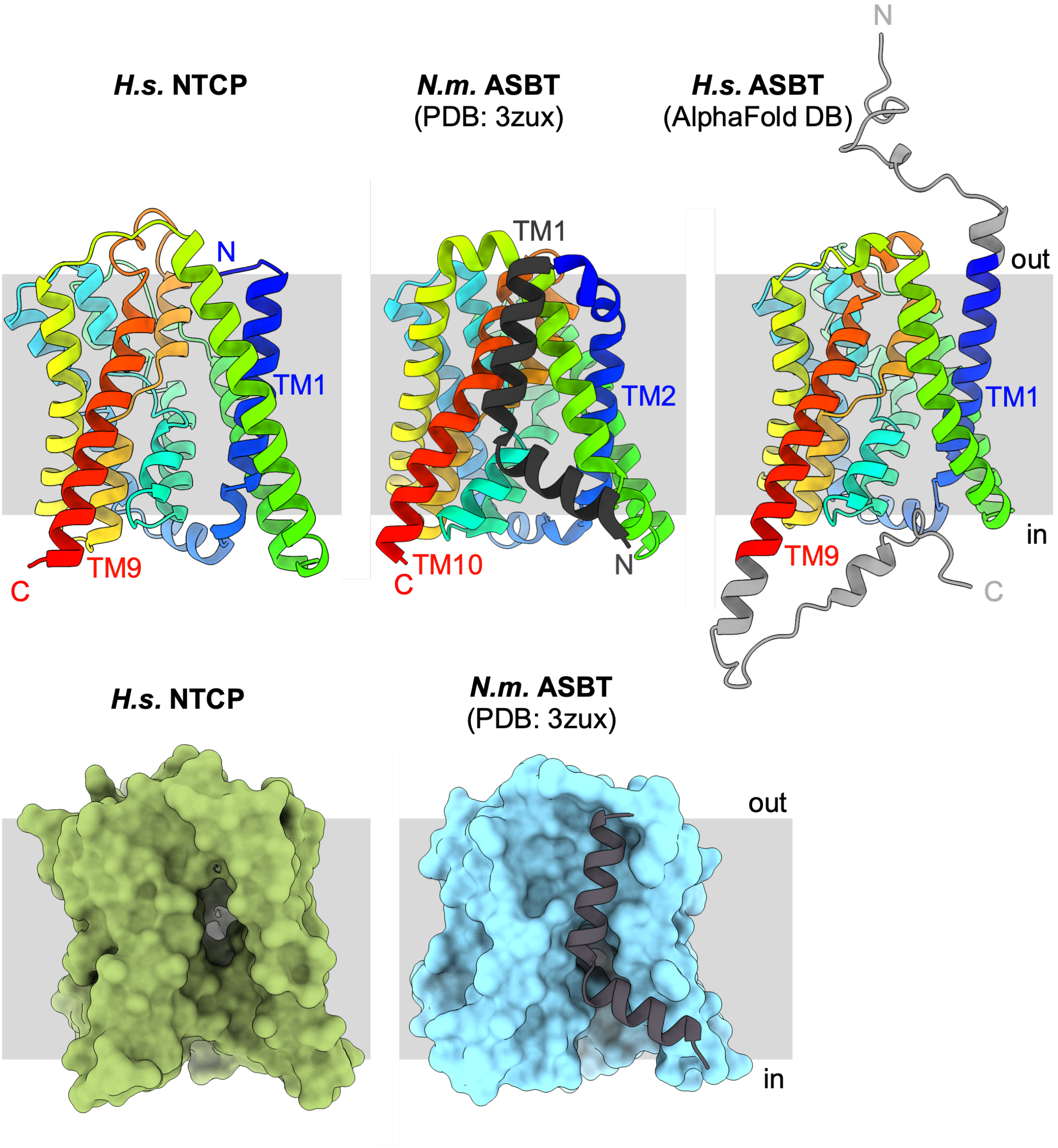

Fig. S5. Comparison of human NTCP structure with the structure of bacterial ASBT homolog and the AlphaFold prediction of human ASBT. The proteins are shown in ribbon representation (top) or as surfaces (bottom). No surface representation is shown of the AlphaFold prediction of human ASBT. The coloring of the TM helices (top) is in rainbow color from blue to red (N to C-terminus). Note that the bacterial ASBT homolog contains an extra TM helix (TM1), which is colored black. This TM helix shields the translocation pathway from the lipid bilayer. Its absence in human NTCP (and presumably also in human ASBT) results in large cavities and a tunnel that presumably serves as the substrate translocation pathway.

Supplementary Information, Figure S6

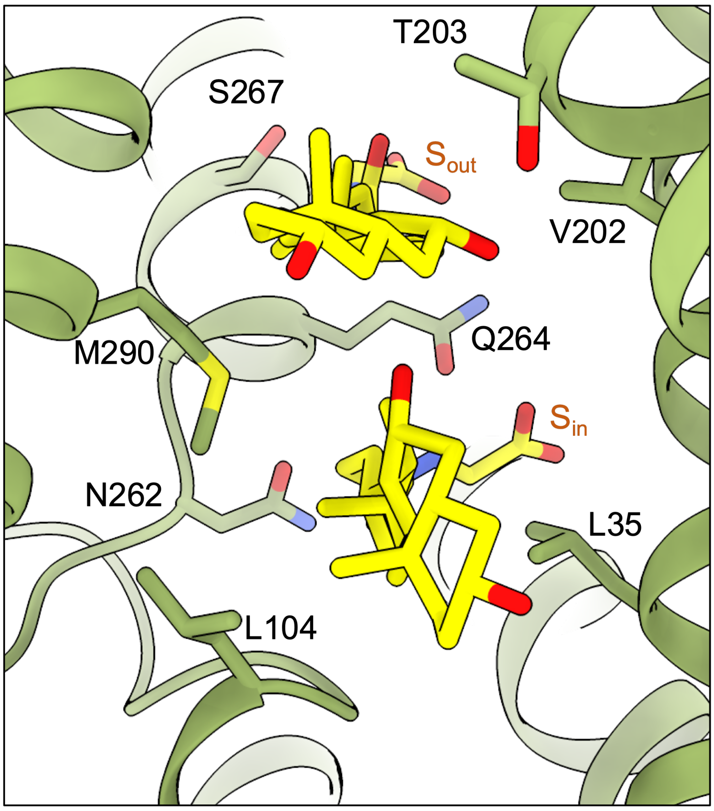

Fig. S6. Close-up of substrate binding site of human NTCP. NTCP is shown in ribbon representation, with two bound GCDC molecules shown in stick representation and colored yellow. Residues within 3.5 Å of the bound substrate are shown in sticks representation.

Supplementary Information, Figure S7

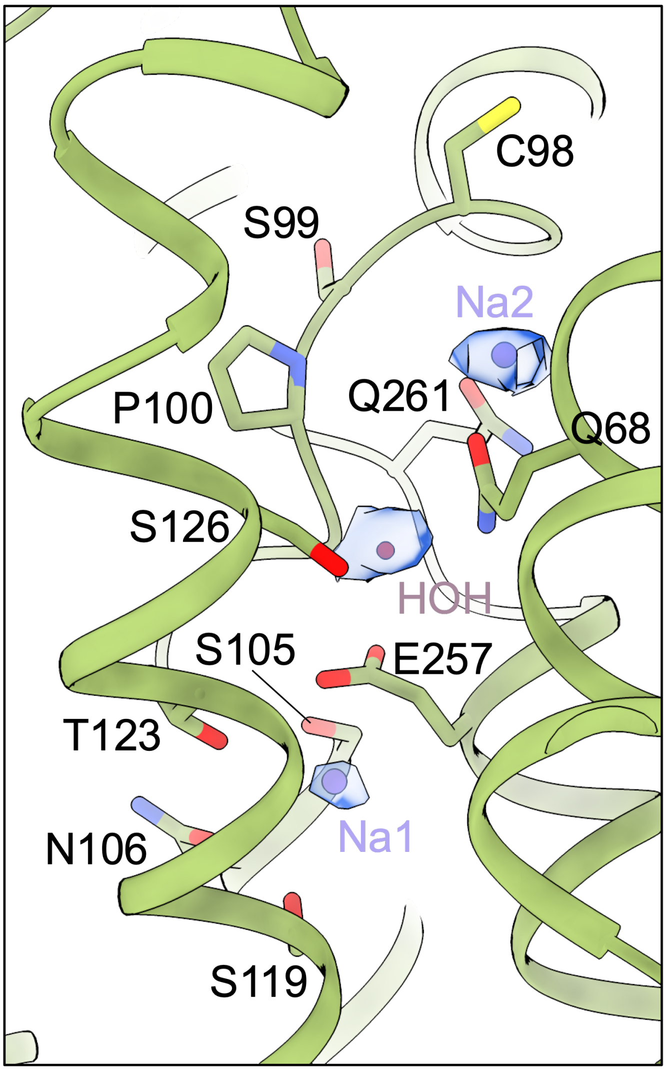

Fig. S7. Close-up of sodium binding pocket in human NTCP. The protein is shown in green ribbon representation, residues within 4 Å of bound sodium (Na1 or Na2) are shown in stick representation and labeled. The EM density map around two presumed Na^{+} ions and a bound water are shown as surfaces at the same threshold.

Supplementary Information, Figure S8

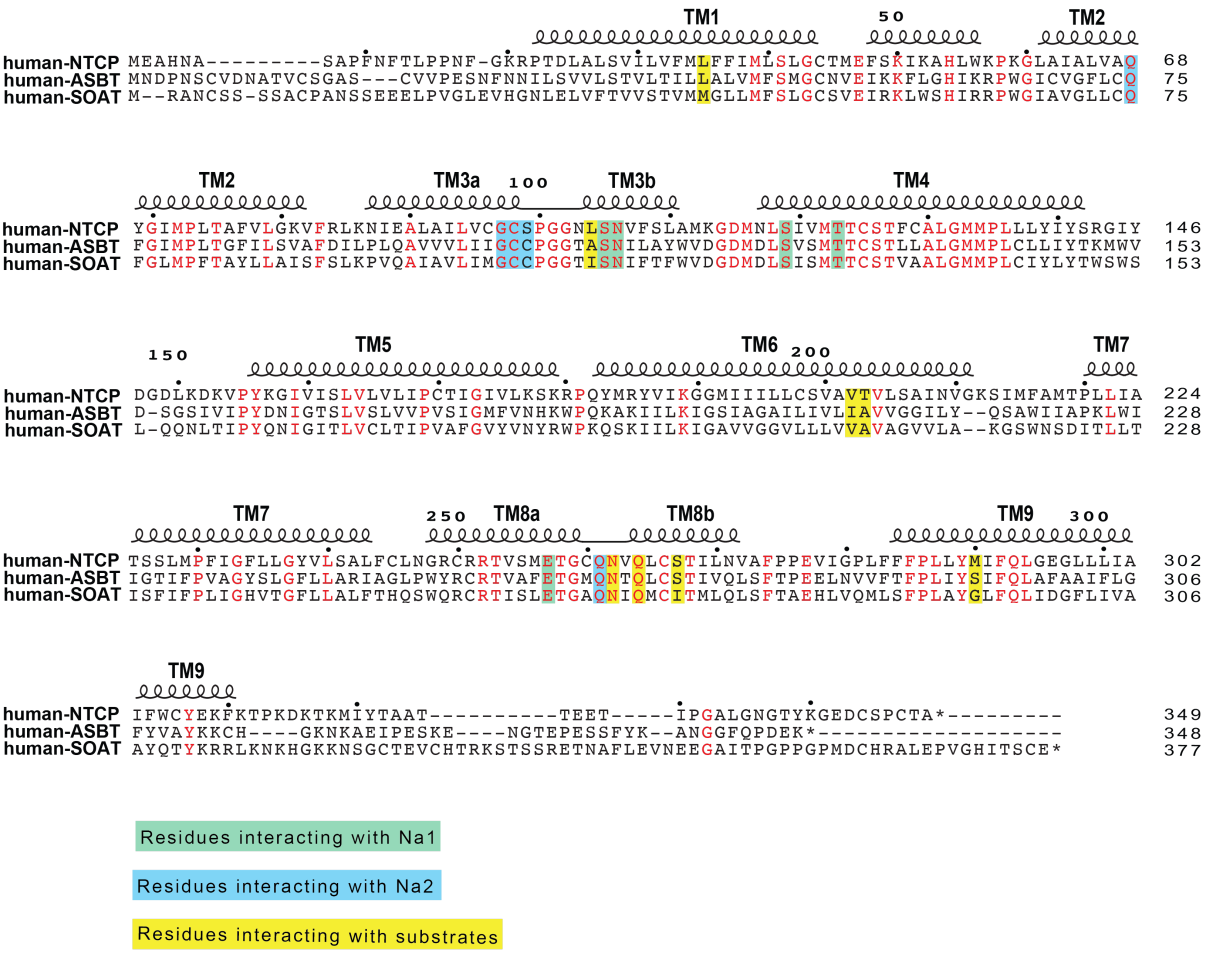

Fig. S8. Sequence alignment of human NTCP, ASBT and SOAT. Structure-based alignment of protein sequences of human NTCP (SLC10A1), human ASBT (SLC10A2), and human SOAT (SLC10A6). Dots above the human NTCP sequence indicate residue numbering. Transmembrane helices are shown as observed in the NTCP structure and labeled. Residues forming the Na1 and Na2 binding sites are shaded green and blue, respectively. Residues interacting with bound substrates are shaded yellow.

Supplementary Information, Table S1.

Cryo-EM data collection, refinement and validation statistics

	NTCP with NTCP_Fab12 and Nb (EMD-15024) (PDB: 7zyi)
**Data collection and processing**	
Microscope	Titan Krios
Voltage (kV)	300
Magnification	130,000
Electron exposure (e^{-}/Å^2)	64
Defocus range (μm)	-0.6 to -2.4
Pixel size in super-res mode (Å)	0.33
Detector	Gatan K3
Movies	13,208
Symmetry imposed	C1
Number of extracted particles	7,663,599
Number of particles after 2D classification	1,517,489
Number of particles in final structure	161,093
Map resolution (Å) at 0.143 FSC criterion	2.88
Sharpening B-factor (Å^2)	-52
Processing software	RELION 4
**Refinement**	
Initial model used (PDB code)	none
Model resolution (Å)	2.5/2.7/3.0
FSC threshold	0/0.143/0.5
Model composition	
Non-hydrogen atoms	6641
Protein residues	852
Ligands	2 Na, 2 CLR, 2 CHO, 1 H_2O
B factors (Å^2) (mean)	
Protein	102.62
Ligand	82.07
R.m.s. deviations	
Bond lengths (Å)	0.009
Bond angles (°)	1.131
Validation	
MolProbity score	1.62
Clash score	8.60
Poor rotamers (%)	0.69
Ramachandran plot	
Favored (%)	97.16
Allowed (%)	2.84
Disallowed (%)	0.00
